# Determination of tip transfer function for quantitative MFM using frequency domain filtering and least squares method

**DOI:** 10.1038/s41598-019-40477-x

**Published:** 2019-03-07

**Authors:** David Nečas, Petr Klapetek, Volker Neu, Marek Havlíček, Robert Puttock, Olga Kazakova, Xiukun Hu, Lenka Zajíčková

**Affiliations:** 1 Plasma Technologies, CEITEC, Masaryk University, Brno, 62500 Czech Republic; 2 CEITEC, Brno University of Technology, Brno, 63800 Czech Republic; 3 Czech Metrology Institute, Brno, 63800 Czech Republic; 4 IFW Dresden, Dresden, 01069 Germany; 5 National Physical Laboratory, Teddington, TW11 0LW United Kingdom; 6 Physics Department, Royal Holloway University of London, Egham, TW20 0EX United Kingdom; 7 Physikalisch Technische Bundesanstalt, Braunschweig, 38116 Germany; 8 Department of Physical Electronics, Faculty of Science, Masaryk University, Brno, 61137 Czech Republic

## Abstract

Magnetic force microscopy has unsurpassed capabilities in analysis of nanoscale and microscale magnetic samples and devices. Similar to other Scanning Probe Microscopy techniques, quantitative analysis remains a challenge. Despite large theoretical and practical progress in this area, present methods are seldom used due to their complexity and lack of systematic understanding of related uncertainties and recommended best practice. Use of the Tip Transfer Function (TTF) is a key concept in making Magnetic Force Microscopy measurements quantitative. We present a numerical study of several aspects of TTF reconstruction using multilayer samples with perpendicular magnetisation. We address the choice of numerical approach, impact of non-periodicity and windowing, suitable conventions for data normalisation and units, criteria for choice of regularisation parameter and experimental effects observed in real measurements. We present a simple regularisation parameter selection method based on TTF width and verify this approach via numerical experiments. Examples of TTF estimation are shown on both 2D and 3D experimental datasets. We give recommendations on best practices for robust TTF estimation, including the choice of windowing function, measurement strategy and dealing with experimental error sources. A method for synthetic MFM data generation, suitable for large scale numerical experiments is also presented.

## Introduction

The point spread function (PSF) concept is frequently used in optical microscopy to estimate the properties of the imaging system and subsequently reduce its impact on the acquired image quality. The PSF represents how the single point on the sample gets blurred by the optical system; the whole recorded image is a convolution of the ideal sample image and the PSF. In the field of Scanning Probe Microscopy (SPM), a similar concept can be used. Though seldom used in routine analysis, the PSF is typically only used in advanced or specialised data processing. Examples include Kelvin Probe Force Microscopy and similar electrostatic forces-based techniques, where the PSF is used to incorporate the effect of averaging the measured signal across the sample and to consider probe shape due to long range behaviour of the electrostatic forces^1–3^. In the field of Scanning Near Field Optical Microscopy it is used to investigate the optical system properties^4^. In Magnetic Force Microscopy (MFM) a similar concept of a Tip Transfer Function (TTF) is used to specify the imaging properties of the probe and thus to receive quantitative information about the sample’s stray field^5,6^. As we concentrate on MFM data processing in the following text we use the Tip Transfer Function terminology, abbreviating it to TTF, however most of the presented algorithms are valid for point spread functions or related transfer functions in other SPM areas as well. The TTF is a simpler and less computationally demanding alternative to full micromagnetic simulations of the interaction between probe and sample which additionally require detailed information about the probe and sample materials and geometry^7–9^. A robust TTF determination which handles the unavoidable presence of noise is considered a major step for a routine application of quantitative MFM (qMFM) and will accelerate its use in numerous problems in nanomagnetism.

The approach used for calculating the transfer function and namely its normalisation in different areas of Scanning Probe Microscopy varies, which makes mutual comparison of TTFs obtained by different groups complicated. Even though TTF reconstruction has been applied successfully to various MFM measurements^5,6,10–14^ the behaviour of the reconstruction methods for MFM data has never been systematically studied. This article aims to suggest suitable procedures and parameters for the TTF calculation, based mostly on numerical studies. In addition, we discuss common pitfalls while calculating the TTF from real data and we present resulting 2D and 3D TTFs obtained on magnetic perpendicular media.

Contrast in dynamic mode MFM is proportional to the probe-sample force gradient, which itself depends on both the probe and sample’s magnetic state. The Tip Transfer Function approach for MFM probe characterisation is based on using a well characterised reference sample for which the stray field pattern is calculable as it consists of magnetic domains of known orientation and possesses magnetic properties, which are measurable or known from literature. One possibility for such a reference sample are thin films with perpendicular magnetic anisotropy, such as the Co/Pd multilayers suggested in ref.^11^. They form striped domains with perpendicular orientation, so called band domains, and knowing the global magnetic properties a pure qualitative MFM image allows estimating the stray field pattern from domain theory. The high perpendicular magnetisation anisotropy of this sample guarantees a magnetisation alignment in the *z* direction (perpendicular to sample plane) greater than 92%, permitting to consider in-plane magnetisation components equal to zero^6^. This calibration sample thus provides a strong and readily calculable stray field above the surface and also a large span of spatial frequencies, which are all helpful in TTF calculation. A calibration measurement of this sample allows to determine the unique TTF which is characteristic to the particular MFM probe.

The estimated TTF can be then used to make quantitative measurements on an unknown sample. Its magnetic properties can be arbitrary, assuming it does not change the magnetic state of the probe. Most commonly the TTF from the reference sample is applied to calculated quantitative values from an unknown sample by convolving the TTF with a simulated charge map and quantifying the deviation from the experimental MFM image^12^. Alternatively, experimental MFM data are deconvolved with the TTF—this corresponds to the classical use of deconvolution for image deblurring^15^. The issue with the latter method is that it is more sensitive to noise and is considerably more erroneous than using a simulated charge map and modelling.

## Theory

In many data acquisition methods the measured signal *m* can be expressed as the convolution of an ideal (actual, sharp) signal *s* with a transfer function (instrument function, point spread function) *p* in presence of additive noise *t*:

1 $$m=s\ast p+t.$$

Here $\ast$ denotes the convolution operator. In the case of SPM this is a two-dimensional convolution since all the quantities are images, i.e. functions of spatial coordinates *x* and *y* (in signal processing the convolution is usually in the time domain).

The convolution with *p* blurs the signal and there is a vast literature dedicated to deblurring, i.e. reconstruction of *s* from *m* and known *p*^15–17^. Techniques have also been developed for blind deconvolution, in which the TTF and deconvolved data are estimated simultaneously, usually in a Bayesian framework^18^.

The problem we face in MFM is that MFM probes are regularly changed (either due to tip wear or to adapt them to the sample under investigation) and each probe possesses individual properties. This therefore requires a calibration routine, which can be routinely applied to each MFM probe. The above mentioned multilayer reference sample possesses a balanced up-down magnetisation structure with smooth boundaries between the two domains and a power spectrum with one prominent spatial frequency. The magnetic surface charge distribution can be estimated by thresholding the measured image, and the stray field can be calculated based on the known saturation magnetisation, film thickness and domain transition width. These and similar samples are thus very suitable for TTF recovery^5,10,11^. Even though the deconvolution is in principle still blind, the ideal signal can be assumed known and we deal with a problem complementary to deblurring, i.e. the derivation of TTF *p* from *m* and the—presumably—known *s*.

Since convolution is commutative, the signal and TTF are interchangeable in (1). However, *s* and *p* differ substantially in character. Many deconvolution algorithms popular in various fields, such as CLEAN^19^ in radio astronomy, are geared towards deblurring and less appropriate in our case. We will describe two approaches suitable for MFM.

## Frequency Domain Filtering

If noise were not present, the problem would be theoretically trivial in the frequency domain. Denoting the Fourier transforms by uppercase letters *M*, *P* and *S*, the convolution $M=SP$ would be inverted by $P=M/S$. In the presence of noise, however, this procedure would greatly amplify the noise at frequencies where |*S*| is small. Therefore, regularisation (or some other constraint) is necessary, leading to formulae for an estimate $\hat{P}$ of *P* of the form

2 $$\hat{P}=\frac{{S}^{\ast }}{|S{|}^{2}+\,{\rm{regularisation}}\,{\rm{term}}}M,$$

where the fraction replaces 1/*S* (superscript star denotes complex conjugation). For given stationary noise and signal, the Wiener deconvolution

3 $$\hat{P}=\frac{{S}^{\ast }}{|S{|}^{2}+|T{|}^{2}/|P{|}^{2}}M,$$

is mean-square error optimal^20^, uppercase *T* again denoting the Fourier transform of noise *t*. However, as the spectral density |*P*|^2^ of the sought function is seldom known, the filter needs to be approximated, for instance by the pseudo-Wiener filter^16,21^

4 $$\hat{P}=\frac{{S}^{\ast }}{|S{|}^{2}+{c}^{2}|T{|}^{2}/|M{|}^{2}}M.$$

This approximation, while reasonable in the case of deblurring, is more questionable in TTF recovery because the frequency content of *M* and *P* can differ substantially. The constant *c* is a scaling factor between *M* and *P*, estimating the ratio ||*m*||/||*p*||, where ||·|| denotes the *L*^2^ norm. We must point out that although formula (4) is usually presented without the factor *c*, a simple dimensional analysis shows that it is then incorrect if *m* and *p* are different physical quantities, which they generally are. The simplified formula (4) with *c* = 1 can only be correct when it is used for simple deblurring where the sharp and convolved image are maps of the same physical quantity.

A simple two-parameter regularisation^22^

5 $$\hat{P}=\frac{{S}^{\ast }}{|S{|}^{2}+\lambda +\gamma |L{|}^{2}}M$$

is also widely used, where *λ* and *γ* are parameters which need to be chosen appropriately (more in section Optimal Regularisation Parameter) and

6 $$|L{|}^{2}=16\,{\sin }^{4}\,\frac{kh}{2},$$

is the squared magnitude of the second-order backward differential operator. Symbols *k* and *h* denote the wave vector and sampling step, respectively. Also its simpler form^23^ corresponding to *γ* = 0 is frequently utilised, as is also done in this work. The pseudo-Wiener filter would then reduce to (5) if ${c}^{2}|T{|}^{2}/|M{|}^{2}=\lambda$, i.e. the measured signal has the same spectral density as noise. A more realistic assumption in scanning probe microscopy is that noise is dominated by independent point noise (|*T*| ≈ const.), leading to the single-parameter pseudo-Wiener filter

7 $$\hat{P}=\frac{{S}^{\ast }}{|S{|}^{2}+\lambda /|M{|}^{2}}M=\frac{|M{|}^{2}{S}^{\ast }}{|M{|}^{2}|S{|}^{2}+\lambda }M.$$

The scaling factor *c* was included in *λ* here.

## Least Squares Method (LSM)

Instead of filtering we can explicitly look for the TTF which minimises the squared norm of the difference between reconstruction and measured data (residual) denoted $R=\parallel m-s\ast p{\parallel }^{2}$. Noting that Fourier transform is unitary, *R* can be equivalently calculated from the frequency components as $R=\parallel M-PS{\parallel }^{2}$, which is useful in practical calculations.

In this formulation *p* is described by a vector of parameters $\xi$ and we seek the vector which minimises *R*$(\xi )$. The two elementary choices of parameter sets are (i) entire *p*, with the value of each *p* pixel being an independent parameter, and (ii) prescribing an explicit function form $p=f(\xi )$. It is advantageous to parametrise the Fourier transform *P* instead of *p* itself, because then ${min}_{\xi }\,\parallel M-P(\xi )S{\parallel }^{2}$ is a straightforward non-linear least-squares problem, which can be solved entirely in the frequency domain. No convolutions are necessary. Fourier transforms are only required to calculate *M* and *S* and then to transform the final fitted *P* back to the spatial domain.

We note that frequency-domain filtering inherently produces *p* images as large as *m* and *s*, even if only a small fraction contains useful information. Thus it may seem that choosing individual *p* pixels as the fitting parameters ($\xi =p$) would lead to number of degrees of freedom comparable to number of data, i.e. pixels of *m*. However, here the *p* image can be chosen to be much smaller; and it should be if the (effective) support of *p* is much smaller than the scan size. The corresponding linear least squares problem is therefore reasonable and better conditioned than frequency domain filtering, although regularisation can be still useful. For the Tikhonov regularisation^24^ the residual is expressed as

8 $$R=\parallel m-s\ast p{\parallel }^{2}+\lambda \parallel p{\parallel }^{2}.$$

By introducing matrix *u* which implements the linear operation *s* *, i.e. discrete convolution with *s*

9 $${u}_{n,k}={s}_{n-k},$$

the convolution can be formally written as a matrix multiplication $s\ast p=up$. Note that all indices and array sizes are two-dimensional multi-indices, i.e. each consists of row and column index. The least squares problem

10 $$\mathop{min}\limits_{p}\,[\parallel m-up{\parallel }^{2}+\lambda \parallel p{\parallel }^{2}\,]$$

is a standard linear problem with normal equations

11 $$({u}^{{\rm{T}}}u+\lambda I)p\equiv ap={u}^{{\rm{T}}}m,$$

where *I* is the identity matrix (depending on the chosen discretisation, which we will discuss in Discretisation and Units, *λ* parameters in (8) and (11) may differ). Matrix *u* is Toeplitz-like. In one dimension it is Toeplitz, in two dimensions it is block Toeplitz, and in higher dimensions it has more deeply nested block Toeplitz structure. Hence *u*^T^*u* and consequently *a* are also Toeplitz-like. Specifically,

12 $${a}_{k,k^{\prime} }={c}_{k-k^{\prime} }+\lambda {\delta }_{k,k^{\prime} },$$

where *c* is the unnormalised cyclic autocorrelation function of *s*

13 $${c}_{k}=\sum _{n=0}^{N-1}\,{s}_{n+k}{s}_{n},$$

where *N* is image pixel dimension. However, *c* is cut to the size of *p* to form the matrix.

The vectors and matrices in the equations can be seen as images. This is obvious for vectors such as *m*, which we introduced as images. Writing out the multi-index *n* as (*n*_*x*_, *n*_*y*_) makes clear that *m*_*n*_ has rows and columns. In the case of matrices, this is enabled by their Toeplitz-like structure. Matrix *u* is formed by elements of image *s*, only organised differently—see Eq. (9). Similarly, matrix *a* is formed by elements of the cyclic autocorrelation image *c*, only organised differently and cut to a region around the origin—see Eq. (12). The addition of *λI* corresponds to the modification of the single pixel at the origin. Representing the matrices as corresponding images is convenient for computer implementation because multiplication by a matrix is then equivalent to image convolution (or correlation for transposed matrices).

Overall, all vectors and matrices involved in the system of Eq. (11), although relatively large, are only of size comparable to *p*, much smaller than the full images. The system can be solved easily using the conjugate gradients^24,25^ method. The only costly operation involved is multiplication of *p*-sized vectors with *a*, which is realised as image convolution, utilising the fast Fourier transform (FFT).

The LSM extends naturally to multiple input images. If the same tip and lift height are used in several measurements (distinguished by index *μ*), the least squares problem (10) becomes

14 $$\mathop{min}\limits_{p}\,[\sum _{\mu }\,\parallel {m}_{\mu }-{u}_{\mu }p{\parallel }^{2}+\lambda \parallel p{\parallel }^{2}\,].$$

The normal equations remain the same, except with *a* being replaced by the sum of matrices corresponding to individual measurements ${\sum }_{\mu }\,{a}_{\mu }$. Similarly in the right hand side *u*^T^*m* is replaced by the sum ${\sum }_{\mu }\,{u}_{\mu }^{{\rm{T}}}{m}_{\mu }$. The images combined in (14) must have the same sampling steps, but their dimensions may differ. We can even construct the normal equations for non-rectangular data, for example images with some regions excluded. The correlations and convolutions entering the equations can be easily computed for irregular regions^26^, unlike Fourier transforms. This can certainly be seen as an advantage. The disadvantage of LSM is larger complexity of implementation compared with frequency domain filtering.

## Discretisation and Units

In practical calculations all the operations written using functions and integrals in Theory and Non-periodicity and Image Boundaries are realised with discrete data. Hence, we replace a function *f*(*x*, *y*) with its sampled values in *N* discrete points *f*_*n*_. The real coordinates are related to the integer indices $x={h}_{x}{n}_{x}$ and $y={h}_{y}{n}_{y}$, where *h*_*x*_ and *h*_*y*_ are the horizontal and vertical sampling steps. We then assume $f(hn)={f}_{n}$, writing formally the component-wise product (*h*_*x*_*n*_*x*_, *h*_*y*_*n*_*y*_) as *hn*.

In this paper we define the discrete norm

15 $$\parallel f{\parallel }^{2}=\frac{1}{N}\,\sum _{n}\,|{f}_{n}{|}^{2}$$

and assume unitary (symmetrically normalised) discrete Fourier transform

16 $${F}_{\nu }=\frac{1}{\sqrt{N}}\,\sum _{n}\,{f}_{n}\,\exp \frac{2\pi {\rm{i}}n\nu }{N}.$$

The discrete convolution of *f* and *g* is the sum

17 $${h}_{n}=\sum _{k}\,{f}_{n-k}{g}_{k}.$$

This is the operation usually provied by numerical software. However, when the integral in convolution (1) is approximated by Riemann sum

18 $$m(hn)\approx \sum _{k}\,s(hn-hk)\,p(hk)\,{h}_{x}{h}_{y}+t(hn),$$

it corresponds in the discrete notation to

19 $${m}_{n}={h}_{x}{h}_{y}\,\sum _{k}\,{s}_{n-k}\,{p}_{k}+{t}_{n}.$$

This is consistent with the dimensional analysis of expression (1) which gives ${\rm{\dim }}(m)={\rm{\dim }}(s)\,{\rm{\dim }}(p)\,{\rm{\dim }}({h}_{x}{h}_{y})$. In MFM the magnetic surface charge map is represented as *H*_*z*_, measured in A/m. Tip-sample force gradient, the quantity imaged by MFM, has unit of N/m. Conventionally it is divided by the permeability of vacuum *μ*_0_, resulting in ‘force gradient’ images in A^2^/m. The TTF then has to be measured in $({{\rm{A}}}^{2}/{\rm{m}})/({\rm{A}}/{\rm{m}}\cdot {{\rm{m}}}^{2})={\rm{A}}/{{\rm{m}}}^{2}$.

Obviously, the factor *h*_*x*_*h*_*y*_ must be applied somewhere when realising the convolution (19) using discrete operation (17), and the same holds for deconvolutions. One option is a meticulous normalisation of all intermediate data, Fourier transforms, norms and other quantities in the computation to ensure they always correspond to sampled continuous physical definitions. The other options, more convenient and thus much more popular, assimilate the factor *h*_*x*_*h*_*y*_ into one of the images. Either the ideal signal *s* is pre-multiplied ${\tilde{s}}_{n}={h}_{x}{h}_{y}{s}_{n}$, allowing to write

20 $${m}_{n}=\sum _{k}\,{\tilde{s}}_{n-k}\,{p}_{k}+{t}_{n}.$$

Or the measured data can be pre-divided ${\tilde{m}}_{n}={m}_{n}/({h}_{x}{h}_{y})$. Or a discrete deconvolution used to obtain a discrete TTF ${\tilde{p}}_{k}$, which is then transformed to sampled physical TTF by post-division ${p}_{k}={\tilde{p}}_{k}/({h}_{x}{h}_{y})$. Images modified for discrete operations can be used either internally or exposed to SPM software users. Pre-division is used for instance in the SigMath environment^6,11^, whereas post-division is used internally in Gwyddion^27^.

As long as they are applied consistently, all the approaches provide physically equivalent final results. Nevertheless, the multitude of options invites misunderstanding and confusion in quantitative comparisons if the intermediate images are exchanged and presented. We strongly advocate for only exchanging and presenting images of sampled physical fields in units described above.

Comparison of regularisation parameter *λ* requires even more care. It has different meanings and different physical units in the individual methods. Furthermore, its numerical value depends on convolution and FFT normalisation and other choices. For the definitions (15) and (16) and post-division of discrete TTF ${\tilde{p}}_{k}$ we can introduce a dimensionless *σ* related to *λ* as follows:

- In regularised filter (5) put $\lambda =\parallel s{\parallel }^{2}\sigma$.
- In pseudo-Wiener filter (7) put $\lambda =\parallel s{\parallel }^{2}\parallel m{\parallel }^{2}{\sigma }^{2}$.
- In LSM normal Eq. (11) put $\lambda =N{K}^{1/3}\parallel s{\parallel }^{2}\sigma$, where *N* and *K* are the respective numbers of image and TTF pixels.

Correct powers of ||*s*|| and ||*m*|| ensure that *σ* does not depend on the magnitude of values in either input image. The quadratic relation between *σ* and *λ* for pseudo-Wiener filter and linear for the other two methods ensure *σ* is approximately proportional to noise to signal ratio. Factor *N* in LSM comes from the number of *u* matrix rows^24^—*σ* then scales approximately with $1/\sqrt{N}$ for all methods (as expected for a least squares problem). The additional factor *K*^1/3^ ensures a more or less consistent *σ* scaling with TTF support size across the methods. Nevertheless, we must note that consistent scaling does not mean *σ* values are directly comparable between different methods and that other choices are possible which lead to different but again consistent scaling.

## Non-periodicity and Image Boundaries

A point which cannot be overemphasised is that real-world samples are not periodic and SPM measurements do not produce periodic images. It was disregarded in the previous section for brevity. However, non-periodicity must be taken into account to have any hope of successful TTF recovery.

**Figure 1:**
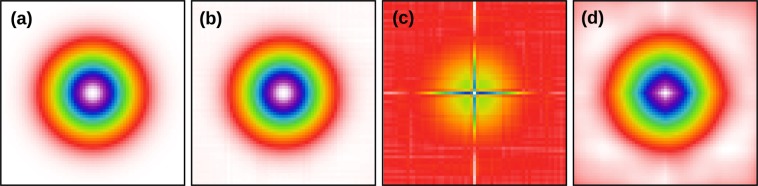
Cross artefact caused by image boundary discontinuities. Transfer functions displayed: (**a**) true; (**b**) reconstructed using the Welch window function and $\sigma \to 0$; (**c**) reconstructed without windowing and the same *σ*; (**d**) reconstructed without windowing and large *σ*—even with *σ* so large that the TTF becomes smeared, the cross-like anisotropy is still clearly present. Note that the colour scale in part (**c**) is adapted to the full value range—the smooth central bump corresponds to TTFs in the other three parts.

Non-periodicity has two basic consequences. First, data measured close to image edges were influenced by sample regions beyond the boundaries of the scanned rectangle. Since these regions were not measured their content is unknown. In deblurring, this causes artefacts close to image edges. However, in the present study it is the least serious of the two problems. The second consequence is contamination of spectra by discontinuities across image edges (which have 1/*f*-like frequency response). The typical result is a ‘cross’ artefact in the reconstructed TTF illustrated in Fig. 1c, which occurs for all TTF calculation methods.

The artefacts can be suppressed by mirror-extending *m* and *s*, which makes them at least *C*^0^-smooth (*C*^*k*^ denoting the class of functions with *k* continuous derivatives). In one dimension a spatially-reversed copy of the signal is placed after it and the new double-sized signal is used instead of the original. In two dimensions this has to be done along both axes, resulting in quadruple-sized images, containing the original plus three differently mirrored copies. Unfortunately, it also means the input data now contain convolutions with *p* in four different positions. Deconvolution then produces *p* averaged over all the positions. If we do not know whether *p* is symmetrical, the result is not useful; whereas if *p* is known to be completely symmetrical, we are better off fitting an explicit symmetrical functional form.

Instead, we utilise windowing^28^. In other words, before the deconvolution using either (2) or (10), both *m* and *s* are multiplied by a window function *w* which smoothly approaches zero at the boundaries. Hence the windowed images also smoothly approach zero at all boundaries and they can be considered periodic. The improvement is illustrated in Fig. 1b.

We remark that both mentioned problems are more serious for two-dimensional images than for one-dimensional signals as comparably larger fractions of data lie close to the boundaries—and it becomes even more serious in higher dimensions as the fraction of hyper-volume lying within a fixed distance from the boundary approaches unity. This also means that windowing has to suppress an increasing portion of data as the dimension *d* grows.

In the spatial domain, the linear relation $m=p\ast s$ holds when *m* and *s* are multiplied by the same constant. The window *w* is a very slowly varying function and the support, or at least the effective support, of *p* is small; even though for instance Gaussian *p* has in principle infinite support. Intuitively, in the corresponding small area the window *w* is almost constant and the relation $m=p\ast s$ is relatively unperturbed by windowing: $mw=p\ast sw$ still holds relatively precisely. Nevertheless, windowing alters the reconstructed TTF. How much and which of the plethora of available window functions should be employed? It is not obvious how window function characteristics studied extensively in harmonic analysis translate to TTF reconstruction.

How much *w* differs locally from a constant depends, in the first approximation, on its derivative (gradient $\nabla w$). If we keep all other settings and only increase the scanned area, the linear dimension of TTF decreases with respect to the linear dimension of the entire area. Denote this ratio of the linear dimensions $\varepsilon$ ($\varepsilon \ll 1$). The *L*^2^ norm of difference of *w* from a constant on TTF support, averaged over image area, is proportional to ${\varepsilon }^{1+d/2}\parallel {\rm{\nabla }}w\parallel$. This is one characteristic of the influence. However, there are other effects to consider. Suppressing more data leaves less information to be utilised in the reconstruction; this is measured by ||*w*||. The window functions also make the data either *C*^0^ or *C*^1^-smooth across the boundary, and this might play a role.

We explored the effect of windowing by numerical simulations in one and two dimensions for several window functions implemented in Gwyddion. They included *C*^1^ Hann, Blackman^28,29^ and Kaiser^30^ ($\alpha =2.5$) windows, *C*^0^ Lanczos and Welch^31^ windows, and *C*^−1^ Hamming^28^ window, which is similar to Hann window but leaves a small discontinuity across the edge.

**Figure 2:**
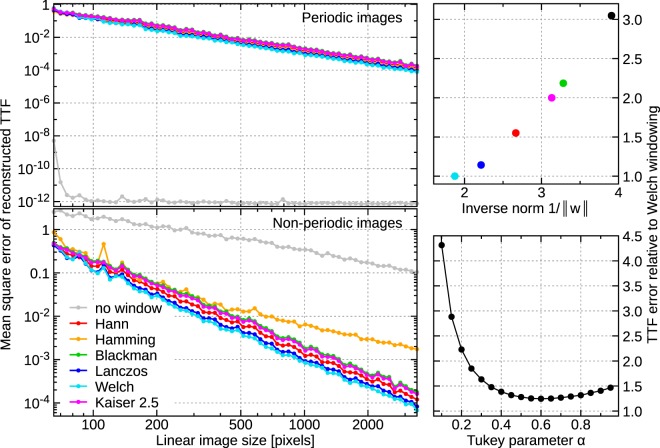
Evolution of mean square error of TTF reconstruction with input data size for several window functions, for periodic and non-periodic noise-free images. The two small plots show the average error relative to the Welch window error for individual smooth window types and the tunable Tukey window.

The results for two-dimensional data (images) are plotted in Fig. 2. They are plotted for both periodic and non-periodic signal *s* consisting of random artificial domain-like images. The true TTF was tetragonal, 21 × 21 pixels and normalised ||*p*|| = 1. In order to isolate the effect of windowing no noise was added and *λ* was set to zero—which is optimal in the noise-free case if windowing is applied (as discussed in Optimum Regularisation Parameter). The TTF was obtained by LSM.

With the exception of no windowing and the *C*^−1^ Hamming window, the results for periodic and non-periodic images were almost identical. Without windowing, the reconstruction is almost perfect in the periodic case. The error is essentially given by the conjugated gradients stopping criterion. However, it is poor for non-periodic images. It should be noted that the no-window curve for non-periodic images does not correspond to the best TTF that could be obtained without windowing. Increasing *λ* would lead to smaller errors. Nevertheless, as Fig. 1d illustrates, even the best case is rather poor.

Any kind of windowing improves the results dramatically, but there are some systematic differences. The scaling behaviour with image size is essentially the same for all window functions which ensure at least *C*^0^ smoothness (the *C*^−1^ Hamming window is clearly inferior). In one dimension the power of $\varepsilon$ is approximately 1.62 while in two it is 2.06. This is not far off the $1+d/2$ powers obtained by considering $\nabla w$ which are 1.5 and 2, respectively. However, when we compare individual windows, the *C*^0^ Welch and Lanczos functions perform best, while the smoother windows perform worse. In one dimension the differences are even more marked. In fact, for all tested windows, the error is strongly correlated to 1/||*w*|| (not $\parallel {\rm{\nabla }}w\parallel$, which is quite similar for all windows) as also illustrated in Fig. 2. Smoothness seems to not matter beyond *C*^0^. The simple Welch window $w(x)=4x(1-x)$ (for $x\in [0,1]$) appears to be a good choice. For a given windowing type the error essentially depends only on the ratio of image linear pixel size to TTF linear pixel size. Figure 2 thus provides an estimate how large a scan area must be to achieve a given error. When the ratio is at least 12–15, which is satisfied for typical image and TTF sizes, the TTF error due to windowing should be around or below 1%.

We also tested the Tukey window function^32^, which is constant on a symmetrical interval around the centre. The interval width is tunable, with wider constant intervals corresponding to more rapidly changing function close to the edges. This window is used in qMFM in the SPM Toolbox^33^. If the parameter was chosen close to the optimum, the performance was slightly worse compared to the Welch window—see Fig. 2. The Tukey window transitions between rectangular and Hann windows as the limit cases. However, the Hann window did not perform particularly well. So there might be space for further optimisation if the tunable window has one of the best performing windows (Welch or Lanczos) as the limit case.

Finally, we remark that in LSM the influence of boundaries can be avoided entirely in a logically consistent manner—at least in principle. If the norm in (8) is calculated only from pixels sufficiently far from the border (farther than TTF size), while the convolution is still done with full-sized images, then no unmeasured data or boundary discontinuities enter the residual *R*. The price is, however, high. The matrix of the corresponding normal equations, although still easy to express formally, has no longer Toeplitz structure; instead, it becomes a general symmetric positive definite matrix. Storage requirements and matrix multiplication complexity increase dramatically. In one dimension this may be still a reasonable trade off, in two and more dimensions the computational cost seems prohibitive.

## Optimal Regularisation Parameter

In the preceding, regularisation parameter was assumed ‘chosen suitably’. When the goal is to measure the TTF corresponding to a particular tip and other parameters (lift height), a suitable choice means small error, expressed as norm of the difference between obtained and true TTF $\parallel \hat{p}(\sigma )-p\parallel$. There are several major contributions to this error:

- Perturbation error, originating from noise and other artefacts in *m* (decreases with increasing *σ*).
- Regularisation error, arising from the difference between regularised and exact problem (grows with *σ*).
- Windowing error, caused by windowing. It can be sometimes avoided, e.g. for strictly periodic data. It can be also traded for an error caused by edge effects but, as shown in section Non-periodicity and Image Boundaries, this is not recommended.
- Ideal data error, caused by our inability to obtain *s* exactly, even with carefully selected and measured samples of perpendicular structures. It will be discussed in section Ideal Image Estimation and Instrumentation Errors.

The first two errors depend strongly on *σ* and we seek a compromise between them. Instead of $\parallel \hat{p}(\sigma )-p\parallel$, which requires to know the true TTF, a surrogate quantity must be optimised. A large number of methods detailing how to choose *σ* have been proposed.

The discrepancy principle^34^ relies on a good estimate of noise in measurement $\delta =\parallel m-{m}_{{\rm{n}}{\rm{o}}{\rm{i}}{\rm{s}}{\rm{e}}{\rm{f}}{\rm{r}}{\rm{e}}{\rm{e}}}\parallel$. We then seek to achieve to match the estimated error, more precisely to achieve $\parallel m-p(\sigma )\ast s\parallel =c\delta$, where *c* is a constant, typically *c* = 2 − 5. The L-curve method^24^ follows from the observation that the parametric curve $(\parallel p(\sigma )\parallel ,\parallel m-p(\sigma )\ast s\parallel )$ is often L-shaped in logarithmic scale, with good compromise between the two errors corresponding to the corner. The generalised cross-validation (GCV) method^24,35^ arises from the requirement that if a single measurement is omitted, it should be predicted well by the remaining data in a good solution. By applying this to all points it is possible to derive explicit formulae for the cross-validation error, expressed using eigenvalues of the matrix of the linear problem. Under certain assumptions, GCV minimises the prediction error $\parallel \hat{p}\ast s-p\ast s\parallel$. Rice’s method seeks instead to actually minimise estimated $\parallel \hat{p}-p\parallel$^36,37^, utilising both a noise estimate and the eigenvalue spectrum.

All these methods work under certain assumptions or in a certain sense, but none work universally. They can suffer a variety of problems, including systematic bias, tricky shape of the minimum, poor behaviour in low-noise cases and instability—and sometimes they can even fail to provide any estimate at all^24,37–41^. One should always verify the suitability of a particular method for specific applications. After many numerical experiments attempting to identify a criterion which works well for this particular application in MFM, we had to ask: Why can a human fiddling with a *σ* slider recognise a near-optimal TTF just by looking at it, without studying eigenvalue spectra or even the residuum? The TTF is not an arbitrary image; it is a localised structure. The compromise we seek is between making it too blurry and too noisy. We observe that even though oversmoothing and noise act very differently—one smears the image, whereas the other increases distant values randomly—both widen the TTF. Therefore, a good TTF should be tight. A suitable tightness criterion should select good *σ* values.

Define TTF function width *w*_ttf_ simply as the standard deviation

21 $${w}_{{\rm{ttf}}}^{2}[\hat{p}]=\frac{1}{|A|}\,{\int }_{A}\,|r{|}^{2}|\hat{p}(r)|\,{\rm{d}}A,$$

where *r* is vector from the TTF centre of mass (or, alternatively, maximum – they usually almost coincide). For the LSM, the integration area *A* is simply the entire $\hat{p}$ image. For frequency domain filtering, best results are obtained if *A* again roughly corresponds to the estimated support of *p*. The course of *w*_ttf_(*σ*) is not sensitive to precise dimensions of *A*. It should be noted that evaluation of *w*_ttf_ is particularly computationally cheap in LSM, because it only involves the small $\hat{p}$ image—no operations with full image-sized data are necessary.

**Figure 3:**
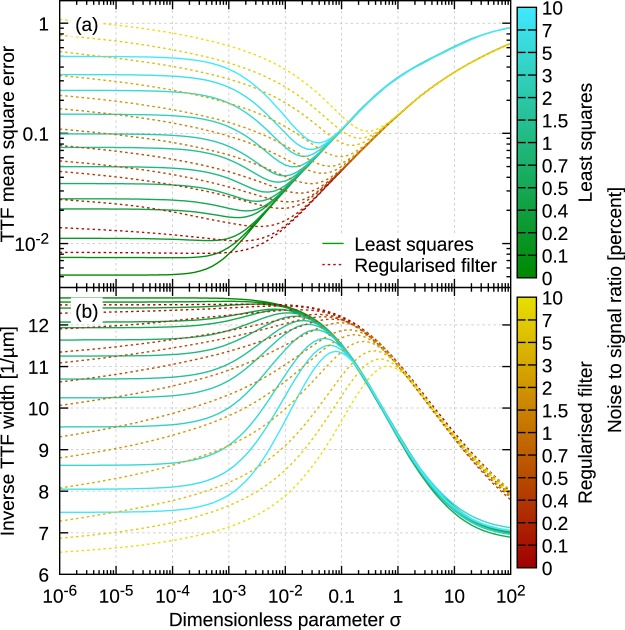
Illustration of *σ* selection criterion based on TTF width: (**a**) actual reconstruction error as a function of *σ*; (**b**) width estimated as *w*_ttf_ as functions of *σ*. Individual curves corresponding to different noise to signal ratios and two different reconstruction methods.

We can now check how well the minimum of *w*_ttf_(*σ*) approximates the minimum of the TTF error $\parallel \hat{p}(\sigma )-p\parallel$. Figure 3a shows the dependency of reconstruction error on *σ* for two algorithms and a set of noise to signal ratios. The displayed curves were obtained using artificial 640 × 640 data for Gaussian TTF and point noise. However, the results were similar for other cases we studied, in particular local scan line errors instead of point noise (as simulated by Gwyddion *Line noise* function) and tetrahedral TTF, which has sharp features and is more sensitive to correct phase than a Gaussian.

Figure 3b shows the evolution of *w*_ttf_(*σ*) (plotted as inverse value for clearer comparison) – and the similarity to Fig. 3a is striking. We can also see that LSM led to systematically smaller errors in the optimal TTF than frequency domain filtering. In Fig. 3a the improvement is between about 20% and a factor of 2. Furthermore, it is evident that LSM behaves considerably better for underestimated *σ* as it is less ill-conditioned. In fact, for low noise there may not be any discernible minimum of $\parallel \hat{p}(\sigma )-p\parallel$ and $\sigma \to 0$ is a also suitable choice.

**Figure 4:**
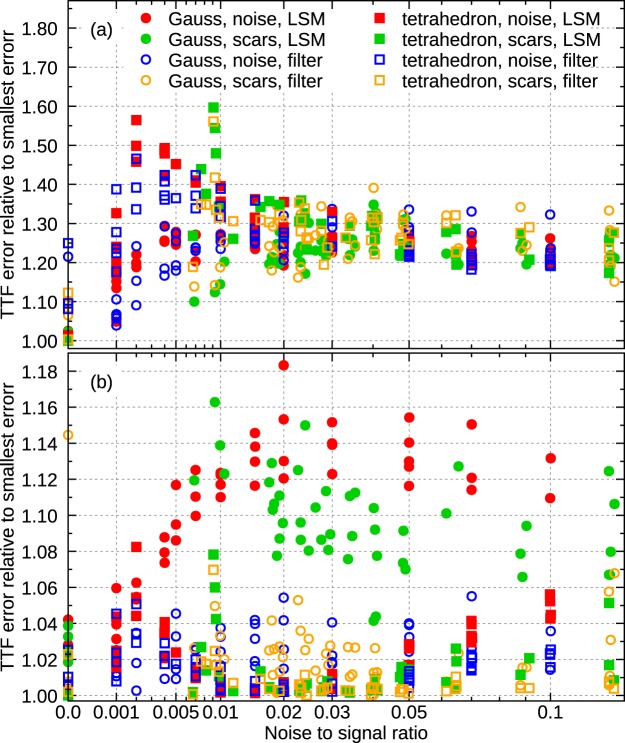
Ratio of TTF reconstruction errors as a function of noise. In part (**a**) *σ* was estimated by finding the minimum of *w*_ttf_ and the plot shows the ratio of TTF error to the minimal error, obtained for true optimal *σ*. The values are evidently scattered; nevertheless, they remain in a narrow interval. Part (**b**) illustrates the improvement when the estimated *σ* is modified by factor 0.35.

In assessing the consequences of replacing the unknown minimum of $\parallel \hat{p}(\sigma )-p\parallel$ with measurable minimum of *w*_ttf_(*σ*), we must therefore consider how much the TTF itself changes with respect to the optimal *σ*—comparing just *σ* estimates would be misleading. Figure 4a shows the ratio of error obtained from the width-based estimate to the smallest error possible for given algorithm:

22 $$\frac{\parallel \hat{p}({\sigma }_{{\rm{w}}{\rm{t}}{\rm{t}}{\rm{f}}})-p\parallel }{\parallel \hat{p}({\sigma }_{{\rm{b}}{\rm{e}}{\rm{s}}{\rm{t}}})-p\parallel }.$$

The ratios are plotted for all combinations of two TTF shapes (Gaussian and tetrahedral), noise types (point and scars) and algorithms (least squares and filtering). Individual values are rather scattered, even though a difference between TTF shapes is observable. The important observation is that the highest ratios are below 1.6 and the typical increase is about 1.3, only a moderate increase.

A closer comparison of *σ* values reveals a small systematic offset between optimal *σ* and position of *w*_ttf_ minimum. The minimum overestimates optimum *σ* slightly. Noting that the cost of overestimation and underestimation differ, the correction factor can be estimated as approximately 0.4. A larger-scale numerical experiment consisting of 23,760 deconvolutions covering wide ranges of image resolutions, noise levels, TTF sizes, and domain widths led to optimum factor of approximately 0.35. The results for *σ*_wttf_ replaced by 0.35*σ*_wttf_ are plotted in Fig. 4b. The error is quite close to minimal for all algorithm and parameter permutations.

The TTF width criterion, as presented above, is therefore a simple and robust way how to optmise the regularisation parameter in TTF reconstruction for all typical MFM image sizes and noise to signal ratios. We should however note that for very large input images with very low noise the method sensitivity is low, i.e. it is not able to differentiate well between a very good TTF and even better one.

## Data Resolution

While measuring the reference sample we need to decide how to set the scan area and pixel resolution of the data. Even if we could search for the best pixel resolution for the TTF reconstruction this would be useless in practice. The goal of TTF estimation is to use it for further calculations, making measurements on some other sample quantitative. Therefore the pixel size of measurement on the multilayer sample used for TTF estimation should be same as real pixel size of the data on unknown sample that we want to quantitatively measure. Fortunately, for most of the probes the TTF is smooth and influence of pixel resolution and scan area (if we can see reasonably the domains on the MFM data) on TTF reconstruction is relatively small.

## Ideal Image Estimation and Instrumentation Errors

Most of the previous discussion was based on numerical experiments with artificial data for which we also knew all the unperturbed images exactly. This allowed isolation of particular aspects and quantification of errors instead of mere estimation. In real MFM measurements, however, the blind deconvolution situation is more messy and there are several image artefacts specific to scanning probe techniques we must deal with.

**Figure 5:**
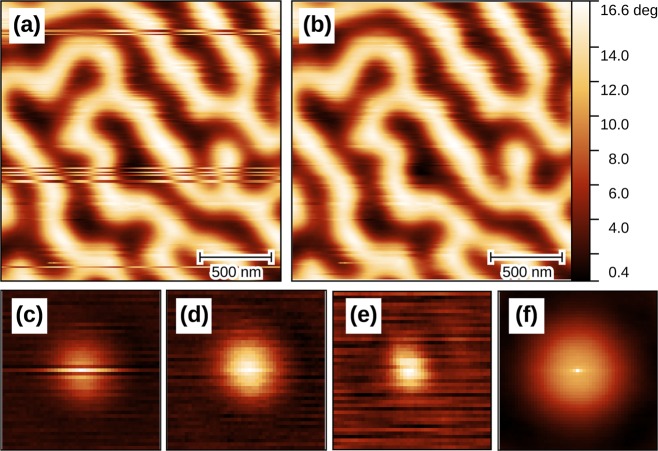
Instrumentation errors and their effect on TTF: (**a**) detail of a MFM phase image with magnetisation switches and other line artefacts; (**b**) the same image with switches corrected; (**c**) strike-through artefact caused by scan line errors; (**d**) strike-through artefact removed by utilisation of uncorrelated images; (**e**) stripe artefacts caused by lift height variation; (**f**) single-pixel spike artefact caused by correlated point noise. The colour scales in parts (**c**–**f**) illustrating individual artefacts are adapted to always match the full data range.

Switching of the tip magnetisation at particular part of the sample is caused by combination of tip magnetisation instability and local magnetic field variations from the sample and is observed relatively often. As a result, the values in entire scan lines are approximately inverted (Fig. 5a). The defect can span several scan lines, or a part of a scan line. It is easy to detect in the data by calculating the discrete covariance of each pair of neighbour lines. Negative values indicate tip magnetisation switch between the two lines. Line inversions must be corrected, otherwise they would wreak havoc on the TTF reconstruction. Interpolation, for instance by solution of Laplace’s equation, is a suitable method. Its Gwyddion implementation gives the result shown in Fig. 5b. A conservative estimate of uncertainty originating from the correction can be based on the fraction of pixels replaced. If all scan lines in the image are replaced by the averages of their neighbours, it results in a distortion in the range 0.5 to 3% under typical conditions. A more specific value can be derived from particular data. By multiplying it with the fraction of replaced pixels we obtain an estimate of additional ‘noise’ introduced by the interpolation.

Even with no inversions present, other line artefacts frequently make the features slightly misaligned between individual scan lines, also visible in Fig. 5. Consequently, each scan line is less correlated to its neighbours than it should be, resulting in a ‘strike-through’ TTF defect, illustrated in Fig. 5c, where a prominent central horizontal stripe is surrounded by suppressed regions. Larger *λ* makes the defect less prominent, at the expense of more blurred result. A surprisingly simple remedy is to utilise two independent scans, one for *H*_*z*_ and one for force gradient. This means to repeat the scan after we see line artefacts present in our data. The line artefacts in the two scans are uncorrelated and thus do not give the horizontal stripe. See Fig. 5d, which was calculated with the same *λ*, only utilising two different scans. Of course, drift must be under control to minimise shifts between the scans. The origin of the line artefacts can be a fault in the feedback loop while measuring the surface topography, similar to the defect observed in purely topographic measurements. It can be also related to the MFM signal measurement in the Lift mode, where two scenarios can lead to line artefacts.

Either the probe stays attached to the surface due to adhesion forces, producing virtually no contrast in MFM data; or the lift height is wrong for the entire scan line, which can be caused by an inaccuracy of the *z*-position sensor. To prevent the former, manufacturers typically retract the probe further from the sample before starting the data acquisition in the Lift mode. For the latter, acquired MFM data are still valid, but acquired closer to or further from the sample surface than requested. Since MFM data decay quickly when moving farther from the sample, the signal in these lines have visually the same shape, but different scale.

In order to simulate the effect of wrong lift height on TTF reconstruction we used a volume MFM dataset and created a virtual experimental data set from MFM signal acquired at different heights. The resulting stripe artefacts are shown in Fig. 5e. The lift height defects can be in principle corrected in the image similarly to magnetisation switches. However, it is much more difficult to mark areas of affected pixels. To avoid excessive correction of experimental data, we should ideally perform a new measurement.

An effect of similar nature can appear for large lift heights, where the signal to noise ratio decreases and the TTF width increases. When there is considerable noise in the measured force gradient, simple thresholding leads to ‘hairy’ edges in the surface charge map. Again, the correlated errors between the images cause artefacts, in this case a sharp single-pixel peak in the TTF centre (essentially representing a *δ*-function). It is depicted in Fig. 5f, calculated from quite noisy data measured at lift height of 250 nm. It is again eliminated by using two scans, a scan with small lift height for the *H*_*z*_ image and the possibly large lift height where we intend to measure TTF for the force gradient image.

**Figure 6:**
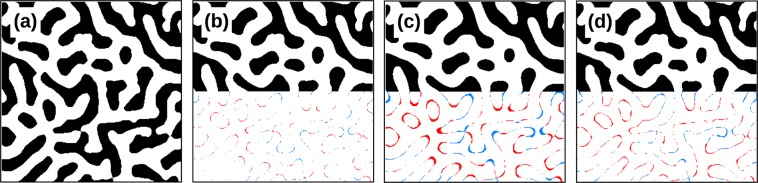
Thresholding artefacts: (**a**) detail of true domain image; (**b**) convolved with Gaussian (width 1/6) and thresholded; (**c**) convolved with Gaussian (width 1/3) and thresholded; (**d**) convolved with Gaussian (width 1/3), 1% noise added, deconvolved and thresholded. Lower halfs of parts (**b**–**d**) show differences from the original binary image, with red and blue representing positive and negative signs, respectively.

This brings us to the final point, the fact that thresholding a force gradient image does not recover the sharp image representing magnetic domains perfectly. Even at small lift heights the shape of reconstructed domain edges is deformed by convolution with TTF. This is illustrated in Fig. 6 using artificial data. Figure 6a displays the actual magnetic domains. The result of convolution and subsequent thresholding is shown in Fig. 6b,c for two different Gaussian TFs, wide 1/6 and 1/3 of domain width, respectively. Differences between Fig. 6b and original are barely noticeable, but in Fig. 6c we can see that finer features were lost. Under typical circumstances, the deformation is moderate and can be to a large degree removed by deconvolving the force gradient image with the obtained TTF and using the result of the deconvolution for thresholding and computation of a second, final TTF.

In Fig. 6d, which illustrates the result of this procedure, we can see the original features were not recovered perfectly, but the distortion was still reduced. Note that the procedure was carried out with 1% noise in the ‘measured’ image. Deconvolution with the first TTF produces artefacts close to image edges (not visible in the selected detail). Fortunately, these regions are suppressed by windowing and even considerable artefact are not detrimental.

## Volume MFM data handling

**Figure 7:**
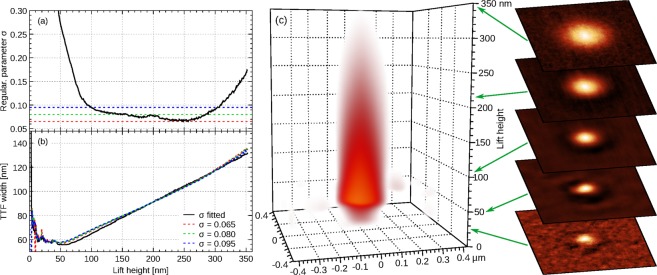
Lift height-dependent reconstruction for volume MFM data: (**a**) evolution of estimated optimal regularisation parameter *σ*; (**b**) TTF width measured using formula (21) for *σ* estimated at each lift height and three fixed values within the range of more or less constant optimal *σ*; (**c**) evolution of TTF with lift height calculated from volume MFM data with slices at selected heights (colour ranges are adapted to full range of each individual slice).

When the procedure of TTF estimation is robust enough for automated work we can use it for more complex dataset processing. This is namely the case for volume data. Present SPMs allow magnetic signal measurements in the whole volume above the sample, with high resolution in all the axes and within reasonable time. Such data can be useful for analysing the stray field decay from the sample surface, e.g. to distinguish between magnetic signal and other probe-sample forces. The volume data interpretation is beyond the scope of this paper, we only demonstrate the performance of TTF estimation algorithm, as shown in Fig. 7.

The first step is to obtain the best *H*_*z*_ from the point of view experimental errors, then apply to all lift heights. The lift height for *H*_*z*_ is selected to be sufficiently high to prevent feedback errors and short range interactions from affecting the data and simultaneously low enough to get good signal to noise ratio. The TTF can be estimated using either constant *σ* or *σ* optimised for each level independently. From Fig. 7a we can see that there is a range in which the estimated *σ* is nearly constant. Therefore, in this region we can use a constant value. However, to cover a wider range of lift heights and still get well reconstructed TTF in each of the lift heights one has to optimise *σ* at each level.

The choice of *σ* has an impact on TTF shape. This is illustrated in Fig. 7b on the plot of TTF width as a function of lift height for different choices of *σ*. A resulting 3D plot of TTFs evaluated at different lift heights is shown in Fig. 7c.

## Conclusion

We considered aspects of tip transfer function (TTF) reconstruction in magnetic force microscopy using measurements on perpendicular domain multilayer structures specially designed for quantitative MFM imaging. With the goal of providing procedures, suitable parameter choices and best practice for measuring the TTF effectively and efficiently, numerical experiments with large sets of simulated data (for which a procedure is outlined) were used to ensure the recommendations hold. Two different classes of deconvolution approaches were studied, regularised frequency domain filtering and solution of least-squares problem. Frequency domain filtering certainly has the advantage of implementation simplicity and while we found it can provide adequate results, the least squares method achieved TTF reconstruction with lower errors and brought other benefits, such as natural extension to multiple input images. We also addressed the tangled issue of regularisation parameter choice for the reconstruction. Noting that in this particular application under- and oversmoothing have the same effect of widening the result, finding TTF with the minimal dispersion was suggested as a simple yet effective regularisation parameter optimisation method. It was verified by a numerical study that it leads to consistently good choices for the regularisation parameter under a variety of conditions covering a range of MFM applications. A robust and automated regularisation parameter estimation has also a benefit that it can be used for volume MFM data analysis, either for cross-checking of data consistency or for estimating the influence of other probe-sample interactions.

Since measurements with a scanning probe bring a specific types of image defects, the effects of most common ones were illustrated alongside measures to avoid them. From analysis of data from numerical experiments and real measurements we can give the following recommendations:

- When possible, measure the experimental data that will be used for TTF estimation twice. Use one of the measurements to simulate the ideal response and another one to estimate the TTF on these data.
- Choose resolution and range of the measurement used for TTF estimation to match the resolution and range of data on the unknown sample on which we want to do quantitative MFM. Use a different resolution only in cases when resulting TTF would be too small or the characterisation sample would be not sufficiently statistically covered.
- Always use windowing when performing the TTF. Based on numerical studies, we recommend the Welch window for TTF reconstruction, for which the error originating from windowing is about 1% or below for typical image dimensions.
- Report TTF and related quantities only as sampled physical fields. We advocate for this even where it might depart from current practice.
- A simple TTF width-based criterion can be used for estimation of the regularisation parameter. Other approaches can be used as well, however be wary when comparing regularisation parameters coming from different methods.

Even if these rules are not exhaustive and there are many experimental conditions that need to be still chosen, following them can signifincantly reduce the TTF errors and potential misunderstandings in TTF interpretation.

## Methods

### MFM Samples and Measurements

Experimental data measured on a reference sample based on Si/SiO_*x*_/Pt(5 nm)/[Pt(0.9 nm)/Co(0.4 nm)]_100_/Pt(2 nm) multilayer are provided within this paper to illustrate the TTF estimation procedure and to point out different instrumentation related errors that we can observe in the measurements. Data were obtained using Dimension Icon microscope with MESP-RC probes in the Lift mode (2D data) or in Force Volume mode (3D data), with typical settings used in routine microscope operation.

### Data Processing

MFM data were processed in Gwyddion (post-2.51 development version)^27^. Artificial data were generated using Gwyddion data processing libraries and modules. Presented algorithms were implemented in C using Gwyddion libraries and FFTW3^42^ and Gwyddion modules and/or library functions.

### Conjugate Gradients

**Figure 8:**
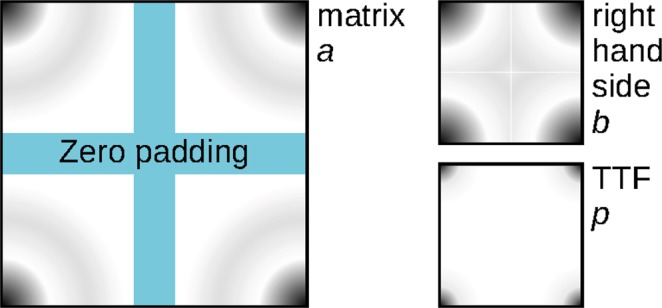
Images representing vectors and matrices in the conjugated gradients procedure.

A simple conjugated gradients procedure is sufficient for the normal Eq. (11). As already noted in section Least Squares Method, the matrices and vectors can be stored as images. We found a consistent FFT-native representation convenient, i.e. during the computation all these images are centred at the origin (top left corner), with large indices *N* − *j* interpreted as negative −*j*. Any zero padding is then done by inserting zeros in the middle—as illustrated in Fig. 8.

Assume *p* is odd-sized with pixel dimension $K=2M+1$ (*K* and *M* being again multi-indices) and the negative index convention. The *p* image then has values *p*_*k*_ where $k=-\,M,-\,M+1,\ldots ,M-1,M$ and the same holds for the right hand side image. The image representing matrix *a* has values *a*_*k*_ with $k=-\,K,-\,K+1,\ldots ,K-1,K$.

The procedure has two stages, initial setup:

1. Apply windowing to *s* and *m* images.
2. Compute image $b=s\ast m$, cut to $2M+1$ size.
3. Compute image $c=s\ast s$ (cyclic autocorrelation), cut to $2K+1$ size.
4. Modify the pixel at origin in *c* by adding *λ*.
5. Start with $j=0$ and ${p}^{(0)}=0$ (if no better initial estimate is available).
6. Compute ${f}^{(0)}={v}^{(0)}=a{p}^{(0)}-b=-\,b$ and ${w}^{(0)}=a{v}^{(0)}=-\,ab$. Images *f*, *v* and *w* all have the same dimensions as *p*.

and iteration:

1. Compute the scalar products $n={f}^{(j){\rm{T}}}{v}^{(j)}$ and $d={v}^{(j){\rm{T}}}{w}^{(j)}$.
2. Iterate solution *p*: $${p}^{(j+1)}={p}^{(j)}-n/d\,{v}^{(j)}$$.
3. Iterate residual vector *f*: ${f}^{(j+1)}={f}^{(j)}-n/d\,{w}^{(j)}$.
4. Check iteration stopping criteria and possibly stop with solution *p*^(*j*+1)^.
5. Iterate *v*: $${v}^{(j+1)}={f}^{(j+1)}-{f}^{(j+1)T}{w}^{(j)}/d\,{v}^{(j)}$$.
6. Iterate *w*: $${w}^{(j+1)}=a{v}^{(j+1)}$$.
7. Increment *j* and repeat.

Matrix multiplication *av* is realised as the image convolution with the corresponding images $a\ast v$, with *v* zero-padded in the middle for FFT.

Since errors tend to accumulate in pixels close to the borders of the solution image, the result can be noticeably improved by increasing the estimated TTF size by 2–3 pixels on each side and then cutting the solution back to the original size.

### Artificial Data Generation

The perpendicular magnetic domain structures used for determining TTF are quite specific. In order to study the behaviour of various algorithms in numerical simulations, an efficient artificial data generation method is necessary. The procedure described below reproduces local features, observable on the scale of TTF support and we implemented it as *Phases* synthetic data module for Gwyddion^27^. It does not reproduce faithfully the global topology, with entire ‘up’ and ‘down’ domains forming single connected components (or at least disconnected components being rare). However, this characteristic is not observable on the scale of TTF and thus not important in the reconstruction.

**Figure 9:**
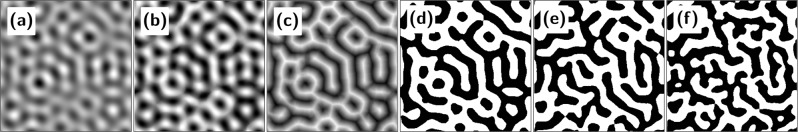
Artificial data generation procedure: (**a**) frequency-domain synthesis; (**b**) local normalisation; (**c**) difference of Euclidean distance transforms; (**d**) final binary image (low frequency spread ${\rm{\Delta }}$*f*); (**e**) final image with moderate ${\rm{\Delta }}$*f*; (**f**) final image with large ${\rm{\Delta }}$*f*.

The procedure combines frequency domain synthesis with morphological processing:

1. A frequency-domain image is formed, containing a ring with amplitude of Fourier coefficients given by function 1/cosh *t*, where $t=(f-{f}_{0})/{\rm{\Delta }}f$. The ring central frequency *f*_0_ and spread ${\rm{\Delta }}$*f* are tunable parameters; the domain widths are proportional to 1/*f*_0_. The phase of Fourier coefficients is assigned randomly.
2. The corresponding spatial-domain image is obtained by inverse FFT (Fig. 9a).
3. Image values *v*_*n*_ are locally normalised to the range $[0,1]$ by replacing them with $({v}_{n}-{m}_{n})/({M}_{n}-{m}_{n})$. Symbols *m*_*n*_ and *M*_*n*_ denote the local minimum and maximum values in circular neighbourhoods of *v*_*n*_. They are calculated by erosion and dilation morphological filters with flat disk structuring elements. The neighbourhood size is proportional to 1/*f*_0_ (Fig. 9b).
4. A binary image is formed by thresholding *v* at level ${v}_{n}\ge 1/2$. The binary image is then thinned. All pixels corresponding to local maxima in *v* are set to 1 in the thinned binary image (this is essentially to compensate their possible removal by thinning). Euclidean distance transform of 0-valued pixels in the binary image produces image *t*^+^.
5. The previous step is repeated with binary image formed by ${v}_{n} < 1/2$ and local minima in *v*, obtaining *t*^−^.
6. Difference image $d={t}^{+}-{t}^{-}$ (Fig. 9c) is thresholded at $d\ge 0$, obtaining a new binary image representing the two domains.
7. Alternating sequential filter with disk structuring elements is applied to the binary image to ensure smoothness of the domain boundaries. The maximum kernel size is again proportional to 1/*f*_0_, but only a small fraction of the stripe width.

The binary image is then used to obtain the stray field image in the same manner as the thresholded image in TTF calculation. The effect of varying frequency spread ${\rm{\Delta }}$*f* is illustrated in Fig. 9d–f.

Efficient algorithms were utilised for all operations in this procedure. If the number of image pixels is *N* and linear domain size is *W* (also in pixels), the inverse FFT complexity is *O*(*N* log *N*). The dilation and erosion filters have complexity *O*(*NW*) if decomposed into operations with smaller structuring elements^43^ and the same holds for thinning. Euclidean distance transform has complexity just *O*(*N*)^43^. Therefore, the operation with largest asymptotic complexity of *O*(*NW*^2^) is the alternating sequential filter^43^ which is, however, only executed with much smaller structuring elements and does not dominate the total computation time.

## Data Availability

The algorithms are available as a part of open source software Gwyddion. All the experimental and generated data are available at http://nanometrologie.cz/ttf/.
